# Implementing Machine Learning Models for Suicide Risk Prediction in Clinical Practice: Focus Group Study With Hospital Providers

**DOI:** 10.2196/30946

**Published:** 2022-03-11

**Authors:** Kate H Bentley, Kelly L Zuromski, Rebecca G Fortgang, Emily M Madsen, Daniel Kessler, Hyunjoon Lee, Matthew K Nock, Ben Y Reis, Victor M Castro, Jordan W Smoller

**Affiliations:** 1 Center for Precision Psychiatry Department of Psychiatry Massachusetts General Hospital Boston, MA United States; 2 Department of Psychology Harvard University Cambridge, MA United States; 3 Harvard Medical School Boston, MA United States; 4 Psychiatric and Neurodevelopmental Genetics Unit Center for Genomic Medicine Massachusetts General Hospital Boston, MA United States; 5 Predictive Medicine Group Computational Health Informatics Program Boston Children's Hospital Boston, MA United States; 6 Research Information Science and Computing Mass General Brigham Somerville, MA United States

**Keywords:** suicide, machine learning, implementation, mobile phone

## Abstract

**Background:**

Interest in developing machine learning models that use electronic health record data to predict patients’ risk of suicidal behavior has recently proliferated. However, whether and how such models might be implemented and useful in clinical practice remain unknown. To ultimately make automated suicide risk–prediction models useful in practice, and thus better prevent patient suicides, it is critical to partner with key stakeholders, including the frontline providers who will be using such tools, at each stage of the implementation process.

**Objective:**

The aim of this focus group study is to inform ongoing and future efforts to deploy suicide risk–prediction models in clinical practice. The specific goals are to better understand hospital providers’ current practices for assessing and managing suicide risk; determine providers’ perspectives on using automated suicide risk–prediction models in practice; and identify barriers, facilitators, recommendations, and factors to consider.

**Methods:**

We conducted 10 two-hour focus groups with a total of 40 providers from psychiatry, internal medicine and primary care, emergency medicine, and obstetrics and gynecology departments within an urban academic medical center. Audio recordings of open-ended group discussions were transcribed and coded for relevant and recurrent themes by 2 independent study staff members. All coded text was reviewed and discrepancies were resolved in consensus meetings with doctoral-level staff.

**Results:**

Although most providers reported using standardized suicide risk assessment tools in their clinical practices, existing tools were commonly described as unhelpful and providers indicated dissatisfaction with current suicide risk assessment methods. Overall, providers’ general attitudes toward the practical use of automated suicide risk–prediction models and corresponding clinical decision support tools were positive. Providers were especially interested in the potential to identify high-risk patients who might be missed by traditional screening methods. Some expressed skepticism about the potential usefulness of these models in routine care; specific barriers included concerns about liability, alert fatigue, and increased demand on the health care system. Key facilitators included presenting specific patient-level features contributing to risk scores, emphasizing changes in risk over time, and developing systematic clinical workflows and provider training. Participants also recommended considering risk-prediction windows, timing of alerts, who will have access to model predictions, and variability across treatment settings.

**Conclusions:**

Providers were dissatisfied with current suicide risk assessment methods and were open to the use of a machine learning–based risk-prediction system to inform clinical decision-making. They also raised multiple concerns about potential barriers to the usefulness of this approach and suggested several possible facilitators. Future efforts in this area will benefit from incorporating systematic qualitative feedback from providers, patients, administrators, and payers on the use of these new approaches in routine care, especially given the complex, sensitive, and unfortunately still stigmatized nature of suicide risk.

## Introduction

### Background

It is estimated that approximately 800,000 people die by suicide each year worldwide, representing approximately 1 suicide every 40 seconds [1]. In the United States, suicide is the 10th leading cause of death [2]. More than 48,000 Americans die by suicide each year, which works out to approximately 129 suicide deaths every day [2]. Encouragingly, from 2018 to 2019, the US suicide rate declined (by 2.1%) for the first time after 13 years of consecutive increases [2]. However, most states did not experience significant decreases, and whether this downward trend will continue remains to be determined [3]. To achieve the ambitious goal of reducing the suicide rate by 20% before by 2025 in the United States [4], it is critical to prioritize the large-scale implementation of existing evidence-based suicide prevention strategies, as well as the development of new approaches.

The health care system is a key setting in which to target suicide risk detection and prevention efforts. Estimates show that upwards of 75% of people who die by suicide pass through the health care system within the year (and up to 50% within the month) before their death [5]. Visits in medical specialty and primary care settings, followed by the emergency department (ED), are the most common leading up to death by suicide; notably, most of these visits do not include a documented mental health diagnosis [6]. In part owing to these findings, increased national efforts have been made to implement standardized self-reported or clinician-rated tools to assess suicidal thoughts and behaviors [7] as well as psychiatric disorders commonly associated with suicide [8]. Indeed, leading national organizations now recommend such validated screening tools for suicide risk as best practice across treatment settings [9].

Although screening questionnaires represent a key component in health care–based suicide prevention, they are not without limitations. For one, such assessments rely on patients to honestly and accurately report on their experiences and symptoms. The myriad barriers to disclosing suicidal thoughts (eg, concerns about involuntary hospitalization or other unwanted consequences and stigma) [10], as well as biases associated with retrospective recall [11], are well established. Indeed, prior studies have shown that up to three-quarters of patients who go on to die by suicide deny any suicidal thoughts in their most recent health care encounter [12]. Second, suicidal thoughts can fluctuate rapidly over short periods [13], posing the possibility that patients may not be experiencing suicidal thoughts at the time of assessment but experience an escalation in suicidal thinking soon after [10]. Third, even brief screening measures take time for patients to complete and providers to administer and review, which may result in suboptimal completion rates and data quality [14], especially in fast-paced treatment settings. Finally, widely used screening measures have evidenced less than ideal diagnostic accuracy for predicting future suicidal behavior, with some research suggesting that even *high-risk* classifications on traditional suicide risk scales are not sufficiently accurate for clinical use [15,16]. It is recommended that validated tools be used in conjunction with clinical judgment to determine suicide risk; however, clinicians are generally quite poor at predicting who will make a suicide attempt [17]. Clearly, there is room for novel approaches to identify patients at risk for suicide in health care settings—not necessarily to replace but rather to complement traditional methods [18].

The development of automated machine learning–based models that use electronic health record (EHR) data (eg, demographic and health information) to predict patients’ risk of suicidal behavior in the future is one such promising approach that has received increasing attention in the literature [19] and media [20]. There are many potential advantages of machine learning models for suicide risk prediction. For example, because such models leverage vast amounts of routinely collected clinical data, they should require little additional provider or patient burden at the point of care and do not rely exclusively on patients to accurately report on their suicidal thoughts. This also makes EHR-based models potentially useful for population-wide suicide risk screening of patients in the health care system, rather than responding only to those individuals who actively self-report suicidal thoughts or whose clinicians who identify suicide risk. Multiple research teams, including ours [21,22], have published results from studies that build and evaluate such suicide risk–prediction models. Overall, findings from this body of work are promising, with algorithms generally demonstrating high levels of accuracy (eg, classification accuracy up to 94% at Mass General Brigham, with similar model performance across 4 other large health care systems) [21,22]. Perhaps most promising is the potential to pair such scalable, low-cost models with evidence-based interventions; for example, patients stratified at the higher end of a suicide risk distribution might be prioritized for more costly and targeted clinical evaluation and monitoring and possibly receive a suicide-focused intervention [23,24]. Along these lines, recent work shows that existing suicide risk–prediction models have sufficient accuracy to be cost-effective if implemented and paired with various evidence-based interventions [25].

Despite the promise of statistical suicide risk–prediction models when used to inform clinical decision-making, with few exceptions [26-28], these efforts have yet to be widely deployed or evaluated in clinical practice. There are many complex practical and ethical questions about how such models—and corresponding clinical decision support (CDS) tools [29] that alert providers with statistical information about patients’ suicide risk and offer decision support contingent on their risk (and potentially other patient factors) at the point of care—would fare in *real-world* clinical settings that currently remain unanswered. For example, when and how frequently would suicide risk–prediction models be updated and providers notified? Would the additional workload associated with responding to automated suicide risk alerts be manageable for providers [27]? Would some machine learning (sometimes referred to as *black box*) models be interpretable or actionable [30]? Questions related to the clinical implementation of such tools also impact the patients whose data are being used to generate predictions—for example, regarding data privacy and communication model results—as well as the health care systems more broadly in which such approaches are used. A key concern, for instance, is that using automated models to identify at-risk patients who are not currently identified via clinical assessments would add more burden to health care systems with already limited resources.

Thus, before widespread clinical deployment, it is critical to partner with stakeholders (eg, frontline clinicians, patients, administrators, and payers) who can help guide such efforts. Recent work in this area has involved collecting self-report survey data from mental health professionals [30] and patients [31] on clinical and operational issues pertaining to automated suicide risk models. A recent study used self-report surveys (n=35) and interviews (n=12) to collect qualitative data from Veterans Affairs (VA) clinicians involved in the recently implemented VA program that uses predictive analytics to identify and provide outreach to veterans at high risk for suicide [32]. However, we are not aware of any published or systematic qualitative research in the form of focus groups or interviews with key stakeholders regarding the clinical implementation of suicide risk–prediction models in non-VA health care settings. Now considered essential to real-world implementation efforts, qualitative methods have the potential to both rigorously and efficiently answer key questions related to both *whether* and *how* [33] we should proceed with implementing new and automated approaches to suicide risk prediction in clinical care.

### Objectives

The aim of this study is to solicit perspectives on the deployment of suicide risk–prediction models in clinical care via focus groups with providers from various departments at a large (>1000 beds, >1.5 million outpatient visits/year) urban hospital. We conducted this study to guide the ongoing development and, ultimately, clinical implementation of a CDS system that provides statistical information from suicide risk–prediction models and corresponding decision-making support to providers at the point of care. Our specific goals are to (1) better understand providers’ current practices for suicide risk assessment and intervention in routine care; (2) determine providers’ perspectives on the use of machine learning models and corresponding CDS tools for identifying and managing suicide risk, including barriers and facilitators; and (3) identify key factors and recommendations to consider in the development of such a CDS system.

## Methods

### Participants

Focus group participants were providers at the Massachusetts General Hospital (MGH) in Boston. Participants were selected via convenience sampling. The senior author (JWS) contacted leadership (eg, chiefs and clinical or training directors) of 4 MGH departments (psychiatry, internal medicine and primary care, emergency medicine, and obstetrics and gynecology) to provide a brief overview of the study and request that they identify providers in their respective departments who might be interested in participating. Leadership from these departments then either circulated (via email or face-to-face communication) the opportunity to individual providers or sent the study team names and contact information of prospective participants, whom the study team then contacted directly. The study team additionally extended several direct invitations to specific individuals within psychiatry and emergency medicine with known interest in suicide prevention or expertise in treating suicidal patients. Of the 60 total providers whom the study team contacted, 56 (93%) agreed to participate (via *implied consent* because a waiver of documentation of informed consent was obtained); of these 56 who agreed, 71% (40/56) ultimately attended a 2-hour focus group. A total of 3 focus groups comprised only primary care providers, whereas the other 7 groups included either psychiatry providers alone or psychiatry, emergency, and obstetrics and gynecology providers. Participants were paid US $250 per hour for the focus groups.

### Ethics Approval

All procedures were approved under MGH Institutional Review Board 2019P001774.

### Procedure

#### Overview

New participants were recruited until no new relevant knowledge was being obtained from the discussions (ie, data saturation achieved) for a total of 10 two-hour focus groups. All 10 groups were conducted from January through March 2020. A total of 7 groups were conducted in person (before the COVID-19 pandemic) and 3 via videoconference (during the COVID-19 pandemic; late March 2020).

#### Baseline Questionnaire

Upon arrival, participants were asked to complete a self-report questionnaire in REDCap (Research Electronic Data Capture) [34], a secure, web-based, electronic data capture tool. This questionnaire consisted of 30 items spanning four categories: demographic characteristics, clinical experience, EHR use (eg, number of automated EHR alerts per week), and current suicide risk assessment practices.

#### Focus Group Discussion

Each focus group ran for 2 hours and was led by 2 to 3 doctoral-level facilitators (KHB, KLZ, and RGF). The study coordinator (EMM) also was present for 8 of the 10 groups, and for 2 groups, 1 or 2 members of the senior author’s (JWS) team observed the discussion. All focus group components after the baseline questionnaire were audio recorded. After the baseline questionnaire, a facilitator met 1:1 with each participant for approximately 10 minutes to review their typical workflow in the EHR, with the goal of gathering information to inform the potential incorporation of a CDS system for identifying and managing suicide risk into workflows.

Next, the majority (approximately 1 hour) of each focus group was spent in an open-ended discussion assessing providers’ current suicide risk assessment and intervention practices and perspectives and attitudes toward incorporating machine learning models for suicide risk prediction (and corresponding CDS tools) in clinical practice, as well as key barriers and facilitators. For this and all group discussions, facilitators used an interview guide (Multimedia Appendix 1) that was developed and refined over several months by the study team and underwent pilot testing in an initial *mock* focus group with volunteers (ie, colleagues not directly involved with the research) identified by the study team.

**Figure 1:**
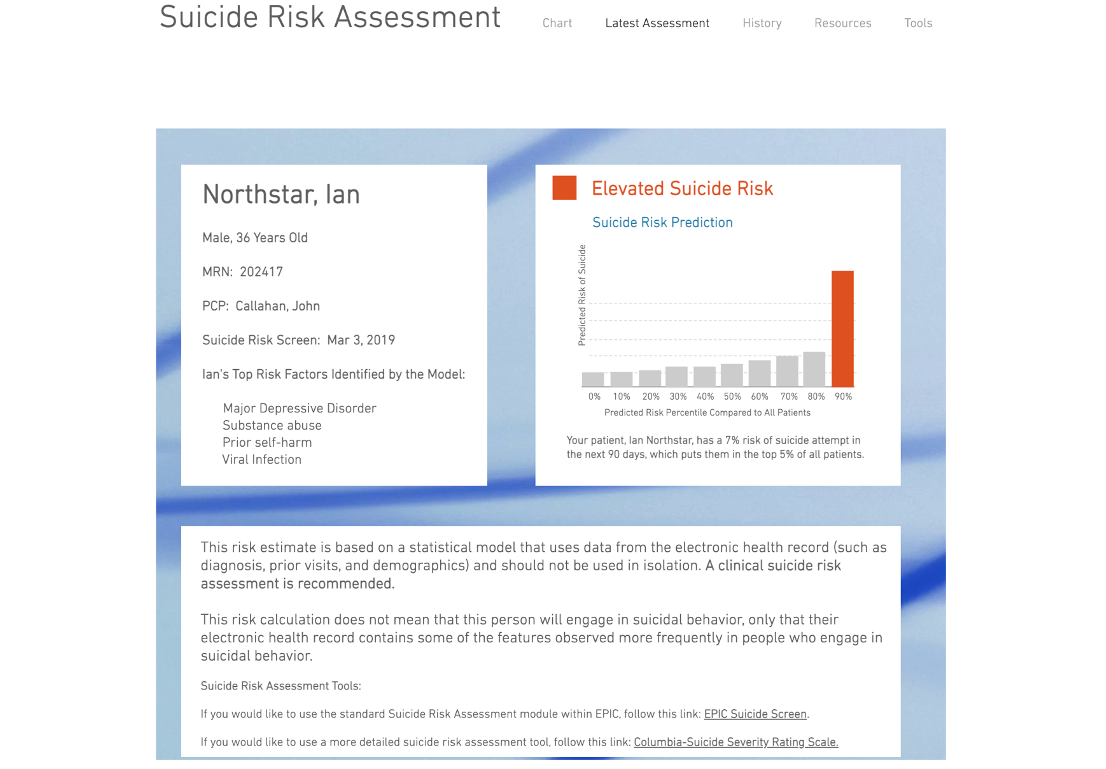
Initial prototype of CDS system for identifying and managing suicide risk shown to participants. Name, demographics, and data shown are for a fake patient. CDS: clinical decision support.

Finally, to elicit more specific recommendations about a potential CDS system for identifying and responding to suicide risk, providers were asked to give feedback on an initial prototype of a CDS tool that communicates statistical information (from a machine learning model using routinely collected EHR data [21,22]) about patients’ suicide risk at the point of care (see the screenshot in Figure 1). Built as a webpage, the prototype consisted of a dashboard displaying demographic characteristics of a mock patient and the patient’s automated *suicide risk score* (from the EHR-based model) as well as their *top risk factors* (ie, strongest predictors in the model) and absolute and relative risks of suicide attempt in the next 90 days, with links embedded to additional resources (eg, clinical suicide risk assessments). This initial prototype did not include clinical recommendations tailored to the suicide risk score. Participants were given 5 minutes to navigate the prototype on their own, after which the interview guide was used to elicit feedback in a brief (approximately 15-minute) group discussion.

### Qualitative Data Analysis

#### Codebook Development

Audio recordings of the group discussions were first transcribed by an external service (TranscribeMe). Then, 3 doctoral-level team members (KHB, KLZ, and RGF) independently reviewed and annotated 2 randomly selected transcripts (for a total of 6 of the 10 transcripts) to inductively derive major and recurrent themes related to key guiding interview questions (Multimedia Appendix 1). Themes were then organized into an initial draft of a codebook, and a comprehensive training manual with coding guidelines was provided to study the staff responsible for coding. Five team members (KHB, RGF, EMM, HL, and DK) then each used the draft codebook to practice coding another transcript using NVivo (version 12) [35] with the goal of identifying new codes to add or irrelevant or redundant codes to delete.

The results of this practice coding and potential new or redundant codes were reviewed, as well as coding issues troubleshooted, in a series of team meetings. Whereas some codes were removed, others were merged or renamed for clarity. The codebook was then finalized to contain 140 unique themes (each with example quotes), organized into the following eight subcategories: (1) current suicide risk assessment practices, (2) current suicide risk intervention practices, (3) attitudes about current assessment and interventions, (4) general reactions and attitudes toward using machine learning models for suicide risk prediction in clinical practice, (5) factors to consider when developing or implementing such models and corresponding CDS tools for suicide risk, (6) barriers and concerns to using such systems, (7) recommendations about system content or format, and (8) recommendations about placement of systems within the EHR.

#### Coding Process

Each of the 10 transcripts was then independently reviewed and coded by 2 of the 3 study coders (EMM, HL, and DK) in NVivo. All coded text was reviewed, and discrepancies were resolved in consensus meetings among the 3 coders and at least one doctoral-level team member who facilitated the focus groups. To account for the fact that some participants were more talkative than others and repeated the same theme across multiple distinct consecutive statements, the same theme was only coded once when the same speaker made the same point multiple times within a 2-minute time span. The final coded versions of each transcript, with consensus achieved on all discrepancies, were used to generate the findings (ie, recurrent, frequently coded individual themes or clusters of individual themes) presented in the *Results* section.

## Results

### Sample Characteristics and Questionnaire Data

The mean age of the participants was 43.1 (SD 12.1; median 40.0) years. Of the 40 participants, 15 (38%) were aged between 25 and 34 years, 7 (18%) between 35 and 44 years, 9 (23%) between 45 and 54 years, 7 (18%) between 55 and 64 years, and 2 (5.0%) between 65 and 74 years. The majority were women (27/40, 68%), with 33% (13/40) identifying as men. Participants were predominantly White (35/40, 88%), followed by Asian (2/40, 5%); of the 40 participants, 1 (3%) was Black or African American, 1 (3%) identified as belonging to >1 race, 1 (3%) preferred not to answer, and 1 (3%) was Hispanic or Latino. Of the 40 providers, 24 (60%) were from psychiatry, 10 (25%) were from internal medicine and primary care, 5 (13%) were from emergency medicine, and 1 (3%) was from obstetrics and gynecology. In addition, of the 40 participants, 28 (70%) were physicians, 10 (25%) were psychologists, 1 (3%) was a social worker, and 1 (3%) was a nurse practitioner. Less than one-third (11/40, 28%) identified as residents or trainees. Providers reported an average of 15.1 (SD 10.9) years of experience treating patients (median 10.5 years); 44% (17/39) of participants reported between 0 and 9.9 years treating patients, 15% (6/39) between 10 and 19.9 years, 26% (10/39) between 20 and 29.9 years, and 15% (6/39) reported ≥30 years. All physicians and psychologists reported working in outpatient settings and 63% (25/40) also in inpatient, emergency, or urgent care settings. All internal medicine and primary care physicians (PCPs) reported working in outpatient settings and just less than one-third (12/40, 30%) also in inpatient or urgent care settings. More than half (22/40, 55%) of the participants reported spending ≥33 hours per week on direct patient care.

More than two-fifths (17/40, 43%) of the participants reported using an EHR for ≥10 years. Just more than one-third (14/40, 35%) of the participants received >20 automated alerts each week, and more than half (21/40, 53%) of the participants reported receiving *too many* automated EHR alerts. It was most common for providers to report feeling *somewhat* burned out (17/40, 43%) by their clinical work, and 30% (12/40) felt *a little bit* burned out. Similarly, providers were most likely to report feeling *somewhat* (21/40, 53%) or *a little bit* (7/40, 18%) burned out by their use of the EHR. Just more than half (21/40, 53%) of the participants reported documenting a patient’s risk for suicide at every visit, followed by 38% (15/40) at less than half of patient visits. The majority (27/40, 68%) of the participants reported commonly using at least one structured or semistructured tool to assess suicide risk: most often, the Patient Health Questionnaire-9 [8] or the structured suicide risk assessment tool embedded in the EHR (both endorsed by 22/40, 55% of participants), followed by the Columbia-Suicide Severity Rating Scale [7] (12/40, 30% of participants).

### Themes From Group Discussion

Both the count of coded themes (across the 10 focus groups) and proportion of groups in which themes were coded are provided in Table 1. The top 5 coded themes in each category are displayed in Figure S1 (Multimedia Appendix 2). Example quotes are presented in Table S1 in Multimedia Appendix 3.

**Table 1:** Counts and percentages of frequently coded themes.

Theme		Times theme was coded	Groups with coded theme, n (%)
**Current suicide risk assessment and intervention practices**			
	Other risk assessment practices (eg, review EHR^a^ and obtain collateral)	56	10 (100)
	Use unstructured clinical interviewing	42	10 (100)
	Use structured or semistructured tools	41	9 (90)
	Consult with colleague, supervisor, or external service	26	9 (90)
	Refer for emergency evaluation or inpatient hospitalization	22	9 (90)
	Assessing or predicting suicide risk is challenging or frustrating	17	7 (70)
	Do not use structured or semistructured tools	17	7 (70)
	Structured or semistructured tools are unhelpful, or clinical interviewing best	13	8 (80)
	Access problem in mental health treatment	13	7 (70)
	Time constraints (associated with thorough suicide risk assessment)	13	6 (60)
	Risk and liability concerns for providers treating suicidal patients	12	6 (60)
	Refer to on-site mental health support	12	4 (40)
	Connect with patient’s current psychiatry provider	11	6 (60)
	Low threshold for consulting psychiatry (includes sending to ED^b^ for evaluation)	10	5 (50)
	Behavioral health team not accessible for consults or supports	9	3 (30)
	Lack of comfort with suicide risk assessment or current practices	8	4 (40)
	Value of on-site (or consulting) behavioral health presence	8	2 (20)
	Structured or semistructured (or mandated) tools are helpful	5	4 (40)
	Develop safety plan or other brief suicide-focused intervention	2	2 (20)
**General attitudes about automated suicide risk–prediction models**			
	General interest or promise, or would trust once implemented	47	9 (90)
	Interest in using tool at point of care or as a BPA^c^	38	10 (100)
	General skepticism, sounds anxiety-provoking, or would not trust	33	9 (90)
	Must outperform clinical judgment or show accuracy before clinical use	33	8 (80)
	Promise in primary care	24	8 (80)
	Promise for identifying high-risk patients who might otherwise be missed	15	7 (70)
	Promise for population-level risk stratification (and resource allocation)	12	7 (70)
	Promise in ED (or psychiatric ED)	9	7 (70)
	Promise for new evaluations and certain types of patients	9	6 (60)
	Little or no promise in ED (or psychiatric ED)	5	4 (40)
**Barriers and concerns**			
	Liability	39	10 (100)
	Low data quality in EHR	19	6 (60)
	Alert fatigue or desensitization to suicide risk alerts	18	7 (70)
	Increase in rates of patients needing emergency evaluations or inpatient beds	18	6 (60)
	Increase access problem in psychiatry or contribute to overall system burden	16	5 (50)
	Other harmful effects for patients (eg, stigma and provider-patient alliance)	15	8 (80)
	Utility depends on interventions that would be triggered	14	5 (50)
	Potential for alert to come during a visit unrelated to mental health	14	5 (50)
	Time constraints (associated with using additional CDS^d^ tool)	10	5 (50)
	How to respond to risk communicated by tool outside of face-to-face visits	9	4 (40)
**Facilitators and specific recommendations**			
	Interest in viewing patients’ predictors or features contributing to risk score	49	10 (100)
	Must have good user interface and user experience	37	9 (90)
	Want to see changes in risk scores over time	34	9 (90)
	Need for standardized workflows for responding and documentation	29	8 (80)
	Information should be available to all patients’ providers	18	7 (70)
	Importance of provider training (including instruction if tool is mandatory)	17	6 (60)
	Should be pushed to, not pulled by, provider	14	7 (70)
	Want more information on how algorithm works or test characteristics	12	8 (80)
	Prompt further assessment with structured or semistructured tools or specific questions	11	5 (50)
	Use tool in combination with clinical judgment	11	5 (50)
	Should distinguish between chronic and time-varying predictors or features	10	6 (60)
	Do not recommend interventions for specific risk scores or features	9	3 (30)
	Information in tool should not be available to others with EHR access	7	4 (40)
	Give recommendations of interventions for specific risk scores or features	7	4 (40)
	Should be pulled by, not pushed to, provider	3	2 (20)
**Other factors to consider**			
	Patients should be able to see information in the tool	21	6 (60)
	Timing of when information is given to provider	20	7 (70)
	Importance of considering whether patients will see this information	18	7 (70)
	Risk-prediction window	16	7 (70)
	Patients should not have access to the information in the tool	14	6 (60)
	Variability in interventions, thresholds, resources, or EHR use across settings	10	5 (50)

^a^EHR: electronic health record.

^b^ED: emergency department.

^c^BPA: best practice advisory.

^d^CDS: clinical decision support.

### Current Suicide Risk Assessment and Interventions

All types of providers most often described using unstructured clinical interviewing (not necessarily in isolation from other assessment methods) to determine suicide risk. Although it was also very common for providers to report using structured or semistructured tools, there were also providers in most groups who stated that they do not use structured or semistructured risk assessment tools. Other common risk assessment practices included reviewing the EHR for relevant historical information, considering known suicide risk factors, obtaining information from collaterals, assessing access to lethal means, and using clinical observation (eg, mental status examination).

Regarding current interventions, providers most commonly described consulting with a colleague or supervisor. It was also common for providers to indicate referring high-risk patients for emergency evaluation or inpatient hospitalization, with a subset having a low threshold for doing so. Providers often acknowledged the access problem in mental health treatment. For PCPs, referring patients to an on-site mental health professional for evaluation or support was often reported, as well as connecting with a patient’s current mental health provider (if they have one). Notably, developing a safety plan or other brief, suicide-focused interventions with empirical support [24] was only mentioned in 20% (2/10) of the groups.

Providers expressed a variety of attitudes about their current practices for assessing and treating suicide risk. Predominantly, participants referred to assessing suicide risk or predicting whether a patient will make a suicide attempt as challenging or frustrating; notably, this was more common among providers in psychiatry and emergency medicine than in primary care. Despite how often structured or semistructured tools are used, it was more common for providers to describe these tools as unhelpful than helpful. Expressing concerns about the risk and liability associated with managing patients at risk for suicide was also fairly common. Some providers also noted the time constraints during visits that can make thoroughly assessing suicide risk difficult, and others expressed an overall lack of comfort with suicide risk assessment and intervention practices. PCPs emphasized both the value of on-site mental health professionals for consultations or support *and* that they are not as available or accessible as would be ideal.

### General Attitudes About Automated Suicide Risk–Prediction Models

The predominant attitude toward the use of automated suicide risk–prediction models in clinical practice was positive, with providers generally highlighting their overall interest in this approach and potential to trust model predictions once implemented in routine clinical care. However, many also emphasized the importance of demonstrating that such a tool outperforms or supplements clinical judgment and is accurate before clinical use. It was less common, but still mentioned in nearly all of the focus groups, for providers to also express varying degrees of skepticism, such as that they would either distrust such a tool or find it anxiety-provoking.

Regarding specific potential use cases, providers (especially PCPs) noted the potential promise to inform clinical decision-making and treatment planning at the point of care (eg, as a best practice advisory) and, to a lesser extent, for purposes of population-level risk stratification and resource allocation. The latter was indicated more often by providers in psychiatry and emergency medicine. Providers were especially interested in the potential for the tool to identify high-risk patients who could be missed via traditional assessments. Providers often noted that the value of suicide risk–prediction models would vary by treatment setting, with primary care as the most optimal, followed by the ED. Less commonly, others explicitly emphasized that such a tool would not be useful in the ED. It was also noted in a few groups that such a tool may have more value during new evaluations than during follow-up visits and with certain types of patients.

### Barriers and Concerns

The single most common barrier to the potential use of automated suicide risk–prediction models in routine care was the implication for liability. For example, many were concerned about being held legally responsible if they decided not to hospitalize a patient who was categorized as high risk and then went on to attempt or die by suicide. It was also fairly common to express concerns about low data quality in the EHR affecting the quality of model output (ie, *garbage in, garbage out*). Another frequent concern was the potential for alerts generated by automated suicide risk–prediction models to increase alert fatigue and, specific to this application, that providers may become desensitized to suicide risk alerts over time.

Providers were also concerned about the potential for such a tool to lead to increased rates of hospitalizing patients in health care systems already facing ED overcrowding and shortages of inpatient beds. The possibility for such a tool to further increase the (currently unmet) demand for outpatient mental health services was often raised, and participants noted that the system must offer additional resources if this tool were implemented. Indeed, some providers emphasized that the usefulness of the tool would depend on what *next steps* (eg, interventions or referrals) are available. Whether there might be other associated harmful effects for patients (eg, stigma) was a concern frequently voiced by psychiatry and ED providers. It was especially common for PCPs (less so other providers) to question the benefits (and actionability) of learning that a patient is at high risk for suicide during an encounter unrelated to mental health (eg, ED visit for a medical reason). Other less frequently noted barriers included time constraints and issues associated with responding to suicide risk alerts if received outside of visits.

### Facilitators and Specific Recommendations

Providers strongly believed that such a tool must have a good user interface and user experience to be helpful. Providers also overwhelmingly described an interest in being able to view the specific predictors (eg, diagnoses, demographic characteristics, and treatment attendance) that contribute to an individual patient being identified as high risk for suicide via the model, with some who encouraged distinguishing between chronic and time-varying predictors (or *risk factors*) within the tool. Providers were also extremely interested in being able to view *changes* in patients’ suicide risk scores over time. The need to establish clear, standardized workflows for both responding to suicide risk predictions generated by the tool and documenting interactions with the tool was also a common theme. Similarly, providers emphasized the importance of receiving systematic training on how to use the tool before it is rolled out clinically, including instruction on whether or not its use is mandatory.

It was more common for providers to state that information about suicide risk generated by the tool should be available to all treaters (across disciplines and departments) for a given patient and less common to indicate that model predictions should not be viewed by certain types of providers (eg, physical therapists and cardiologists) and other medical or administrative staff (eg, medical assistants and receptionists). It was generally preferred that suicide risk scores (or alerts) be *pushed to*, rather than *pulled by*, the provider. Some requested having information available within the tool on how the algorithm works and key test characteristics (eg, sensitivity and specificity). It was fairly common for providers to suggest that the tool offer specific follow-up questions to ask, or standardized scales to use, for additional (clinical) suicide risk assessment. However, it was slightly more common to express the view that the tool should not recommend specific interventions based on risk score or primary predictors as opposed to the view that the tool *should* recommend specific interventions. Non-PCPs (eg, psychiatry or ED) often emphasized that the tool be used in combination with (not as a substitute for) clinical judgment.

### Other Factors to Consider

Other factors that participants recommended be considered included the timing of when statistical information about suicide risk is delivered or made available to providers (eg, before, at the start of, or during visits, particularly given the possibility of no-shows) as well as the temporal window used for model predictions (eg, risk over the next week, month, or year). Providers generally believed it would be vital to decide early on whether patients will have access to their suicide risk scores, as this could have implications for what information is included in the tool and wording of the text. It was more common for providers to express the view that patients should (vs should not) have access to model predictions. Providers also noted that there is wide variability across treatment settings in interventions available, conventions of EHR use, and relevant risk thresholds (eg, to warrant categorizing a patient as *high risk* for suicide) and that taking this into account would be important to consider when developing a tool intended for use across the health care system.

## Discussion

### Principal Findings

Interest in machine learning models for suicide risk prediction has proliferated in recent years. The potential to use such models and corresponding CDS systems in routine care is compelling; however, before incorporation into practice, it is critical to partner with key stakeholders who can offer the perspectives and feedback necessary for successful clinical implementation. This focus group study with hospital-based providers revealed several key areas of findings that have direct implications for ongoing and future development and deployment efforts.

First, providers were not satisfied with currently available suicide risk assessment methods. For example, despite the fact that most providers endorsed using structured or semistructured measures of suicide risk, existing tools were more often described as not particularly helpful rather than helpful. However, it is important to consider that this finding in the context of providers also tended to report feeling burned out by their clinical work, posing the possibility that negative attitudes toward current assessments are driven in part by burnout about their job in general (rather than only a specific dislike for current tools). Although unstructured clinical interviewing was generally viewed as the best available method for determining suicide risk, recent research suggests that providers tend to be quite poor at predicting the risk of suicidal behavior [17]. Indeed, many providers referred to how challenging and frustrating it can be to determine a patient’s risk for suicide. Taken together, these findings underscore the clear opportunity to improve upon existing, traditional methods of suicide risk assessment.

Encouragingly, reactions to incorporating machine learning models for suicide risk prediction in clinical practice were positive overall. These approaches appeared especially promising to providers if used to identify and notify providers about patients at the high end of the suicide risk distribution either at the point of care (eg, to inform clinical decision-making) or for purposes of population-level resource allocation, meaning that those identified as higher-risk would be prioritized to receive suicide-specific interventions or more costly or difficult-to-access treatment and follow-up. Our recent work suggests that suicide risk–prediction models may have especially good accuracy when identifying patients at both the very high *and* very low ends of the risk distribution [36]. Given this, it may be worthwhile to frame and leverage these models such that they are also clinically useful for providers seeing patients who are very *unlikely* to go on to make a suicide attempt (and thus may not require further intervention).

Some providers also expressed skepticism, a lack of trust, or anxiety about the potential use of automated suicide risk assessment models in clinical practice, with liability as the single most discussed concern about using these methods in practice. These findings suggest the importance of partnering with individuals and organizations who specialize in medical risk management, as well as with other relevant legal and payer stakeholders, as these tools are brought closer to clinical use. Overarching concerns about liability may be somewhat mitigated by emphasizing both the importance of using model predictions in conjunction with clinical judgment and that high-risk scores should be interpreted in the context of all other available information to make treatment or discharge decisions. Broadly, developing standards of care accompanied by systematic psychoeducation, training, and protocols to accompany such CDS tools for the providers who will ultimately use them may be critical to address concerns about liability and foster buy-in and promote confidence in their use.

Another concern was that alerts from such a CDS tool that incorporates suicide risk–prediction models would be disruptive to providers’ workflows and result in alert fatigue. To maximize the clinical utility and cost-effectiveness of machine learning–based suicide risk models, empirically derived risk thresholds that balance the relative value of avoiding false-negative and false-positive errors [25,27] must be established. Indeed, recent work in the Kaiser Permanente health care system suggests that the degree to which suicide risk models add to clinical workloads depends heavily on the risk threshold selected, along with the approach for responding to these alerts [27]. Such thresholds may also vary by setting; for example, in psychiatry, a higher threshold may be preferred to reduce the number of false positives.

An overarching concern was that EHR-based suicide risk models and corresponding CDS tools would result in an increased burden on already overburdened health care systems. This could happen via either increased rates of sending patients to the ED for further evaluation or inpatient hospitalizations (possibly in part driven by provider anxiety and liability concerns) or more demand for outpatient specialty services. This is consistent with the fact that providers noted already having a low threshold to section patients and problems accessing mental health treatment. Moreover, as some providers stated that the tool’s usefulness would depend on what interventions are available to them, health systems may benefit from determining the approximate number of *newly identified* (via the model) patients who will require interventions or referrals (eg, through simulation studies) and whether current resources can handle potential increases in demand. Developing new resources (eg, urgent care clinics and suicide-focused treatment options) or partnering with organizations outside the hospital system (eg, crisis services and referral programs) may be needed before clinical implementation.

In addition to practical concerns, providers also overwhelmingly voiced interest in viewing the individual predictors or risk factors that contribute to a patient’s elevated risk score. Providers suggested that this information may also guide next steps for intervention. This suggestion is in line with recent research [30] showing that providers may be less likely to use the information from suicide risk–prediction models if the relevant clinical features are hidden *or* they do not view the provided features as intuitively connected to suicide risk. Thus, there appear to be both pros and cons for sharing information on the specific features in these models. Displaying these features could also lead to misinterpretations about causality (eg, the assumption that if a provider targets a certain feature, suicide risk will decrease), which would not accurately reflect the complex, interactive nature of features in machine learning models.

Providers also raised various complicated yet important-to-consider issues such as the timing of when risk alerts would be delivered to providers (and corresponding concerns about, eg, receiving a *high-risk alert* outside of a visit or when a patient does not attend a scheduled appointment) and risk-prediction windows. Although there is great interest in improving short-term suicide risk prediction (eg, over hours, days, or weeks) [37], the degree to which EHR-based models accurately predict short-term risk and are sensitive to change may be limited. In the future, in addition to the clinical questionnaire data that some models incorporate [38], combining EHR-based data with fine-grained *real-time* data (eg, from smartphones or wearable devices [39]) that may capture more acute increases in risk may help meet this need. In the meantime, balancing clinical utility with the accuracy of different risk-prediction windows is critical.

Providers had mixed views on who should have access to the results from automated suicide risk–prediction models. Some felt it was important for all treaters of a given patient to access these results, whereas others thought there may be certain providers for whom such information would be irrelevant or unnecessary and who, for example, may lack training and experience with approaching the issue of suicide sensitively. Providers were also mixed as to whether patients should be able to receive this information. Given the increasing momentum of the OpenNotes movement [40], including within the mental health domain [41], it seems likely that over the long term, we can expect more EHR information to be available to service users. Thus, partnering with patient stakeholders [31] to develop protocols for transparent, sensitive communications about suicide risk, including key contextual information (eg, how to interpret being in a *high-risk* category) and language, may also be strategic.

### Limitations

There are several notable limitations of this study. First, our findings reflect only the views of providers from a single academic medical center in the Northeastern United States and may not generalize outside of this clinical context. Second, the vast majority of participants were White, and thus, the findings of this study may not generalize to providers who identify as minorities. Third, convenience sampling was used for recruitment, which may also limit generalizability; for instance, it is possible that participants were more enthusiastic about suicide risk–prediction models because some were selected for their known interest or expertise in suicide prevention. Fourth, we did not systematically assess providers’ prior experience with suicide risk–prediction models, which may influence attitudes toward these tools. We were also unable to systematically disaggregate theme frequency by provider type or clinical setting, which may obscure important differences in perspectives on using suicide risk–prediction models in practice.

### Future Directions

There are a few key priorities for future investigation. First, as the development of CDS tools for suicide risk prediction is ongoing, qualitative data must be collected both on more fully developed prototypes and during or after initial clinical implementation of such tools. Our emphasis in this study’s series of focus groups was on general attitudes and perspectives toward such approaches, whereas future work will focus on tool development, refinement, and implementation. Second, given the potential scalability of suicide risk–prediction models, future work must include the full range of providers working in a broader range of urban and more rural hospitals nationwide, as well as community health centers and other nontraditional health care settings (both psychiatric and other medical settings, particularly given the enthusiasm from providers here about potential use in primary care). Relatedly, as noted earlier, patient stakeholders must be included in the process of developing and implementing these new tools. More work is also needed to determine how health care systems and individual providers may best leverage and combine information from machine learning models and traditional clinical assessments [18,36]. Finally, given recent work suggesting the potential for suicide risk–prediction models to exacerbate existing racial or ethnic disparities [42], upcoming work should explicitly probe providers’ (and other stakeholders’) perspectives on how to address this critical issue before clinical implementation.

### Conclusions

Deploying virtually any new tool in practice has challenges, but given the complex, sensitive, and unfortunately still stigmatized nature of suicide, it is especially vital that researchers working in this area involve the full range of stakeholders in each stage of the implementation process. Overall, providers in the current qualitative study were dissatisfied with current suicide risk assessment methods and were open to the use of a machine learning–based suicide risk identification and management system to inform their clinical decision-making. This work highlights several key potential barriers and facilitators to be addressed in ongoing and future efforts to develop and implement such models and corresponding CDS tools in routine care and thus potentially better prevent patient suicides within health care systems.

## Appendix

Multimedia Appendix 1 Interview guide (abridged): prompts to guide group discussion.

Multimedia Appendix 2 Top 5 frequently coded themes in each category.

Multimedia Appendix 3 Example quotes for frequently coded themes.

## Abbreviations

CDS: clinical decision support
ED: emergency department
EHR: electronic health record
MGH: Massachusetts General Hospital
PCP: primary care physician
PI: principal investigator
VA: Veterans Affairs
