# Supplementary material for: Implementing Machine Learning Models for Suicide Risk Prediction in Clinical Practice: Focus Group Study With Hospital Providers
Source: JMIR Form Res. 2022 Mar 11;6(3):e30946. doi: 10.2196/30946 (PMC8956996; doi:10.2196/30946)
Supplement: Multimedia Appendix 1 [file formative_v6i3e30946_app1.docx]

**Interview Guide (Abridged):**

**Prompts for Group Discussion**

[Orientation/background provided]

*Note that open-ended probing questions were used to follow each prompt as needed.*

- “How do you currently determine or assess patients’ suicide risk in your practice?”
- “How do you currently respond to indications of patients’ suicide risk (whether based on your own clinical assessment or a notification)?”
- “What are your general impressions about the approach of using data in the EHR to generate information about suicide risk?”
- “*Who* should access or receive the results from this clinical decision support (CDS) system?”
- “What types of recommendations should the system provide?”
- “Could you imagine yourself using the information provided by a CDS system like this, and if so, how?”
- “Do you see any barriers to using the information from this type of CDS system and if so, what are they?”
- “*How* would you want to access such a CDS system?”
- “If the information from this CDS system were to be deployed at the point of care, what would be the best point during your clinical workflow to see it?”
- “If deployed at the point of care, how could such a CDS system impact your clinical workflow?”
- “What information should be included in such a CDS system?”
- “What are your thoughts on whether/how patients should be able to access the information provided by the CDS system?”

[Prototype of CDS tool presented]

- “What are your first impressions of this?”
- “What about this might be helpful to you?”
- “What about this might not be helpful to you?”
- “What do you think about the information on suicide risk provided?”
- “What do you think about the recommendations and resources provided?”
- “What do you think about the language or wording of the text used?”
- “What do you think about the amount and type of information provided on how the algorithm is operating?”
