# Supplementary figures and images for: Implementing Machine Learning Models for Suicide Risk Prediction in Clinical Practice: Focus Group Study With Hospital Providers
Source: JMIR Form Res. 2022 Mar 11;6(3):e30946. doi: 10.2196/30946 (PMC8956996; doi:10.2196/30946)

**Multimedia Appendix 2.** Top-five frequently coded themes in each category.

| 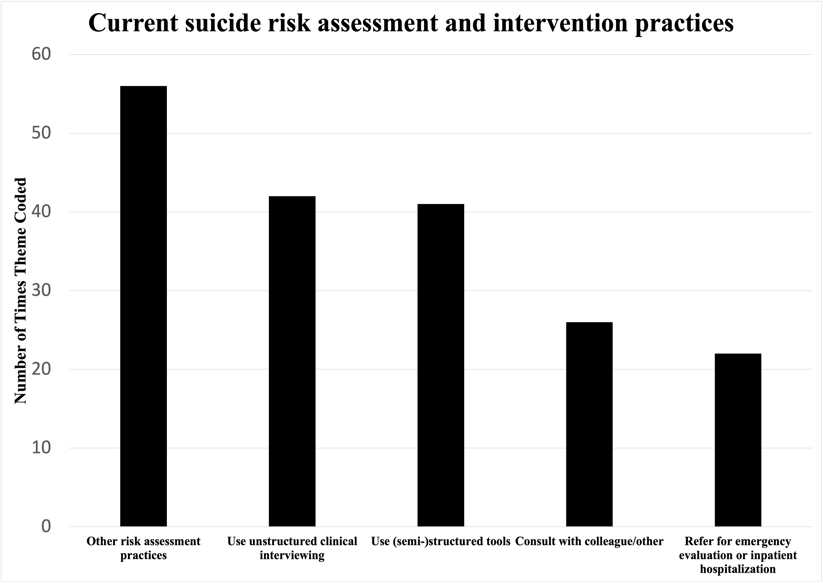 | 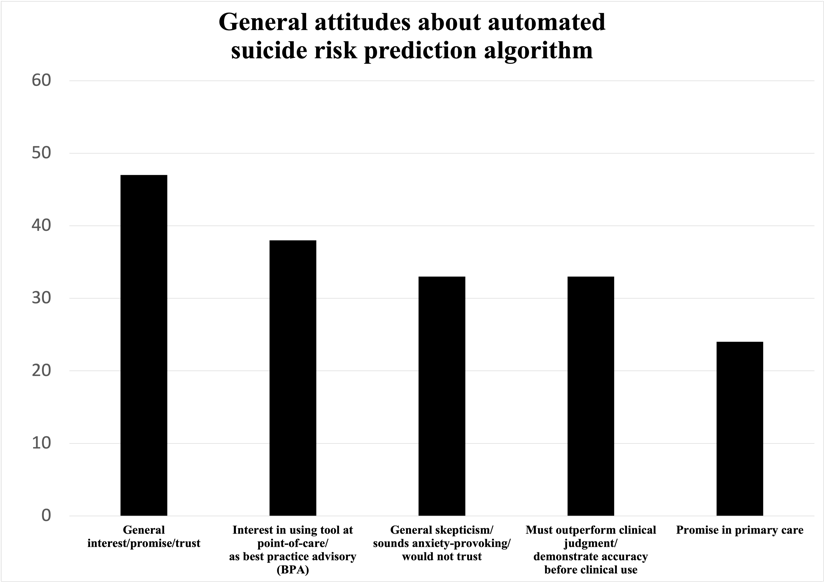 |
| --- | --- |
| 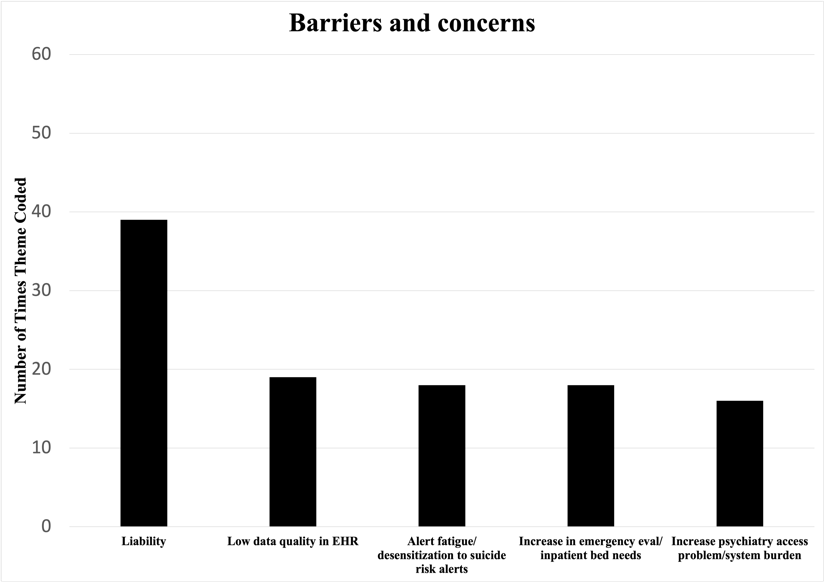 | 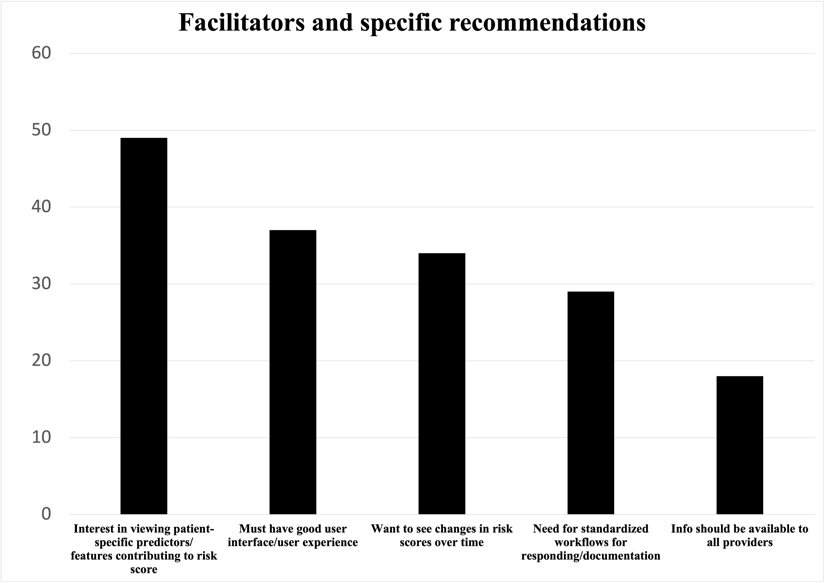 |
| 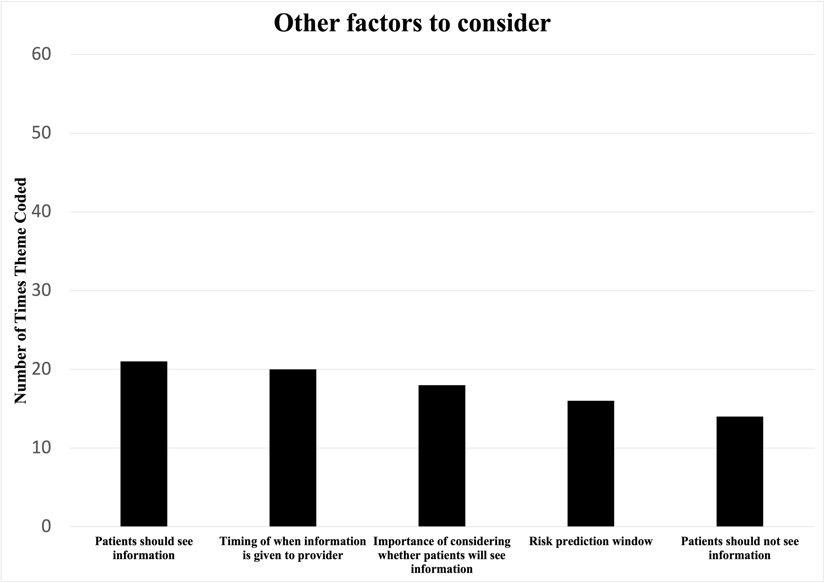 |  |

Supplement: Multimedia Appendix 2 [file formative_v6i3e30946_app2.docx]
