# Supplementary material for: Implementing Machine Learning Models for Suicide Risk Prediction in Clinical Practice: Focus Group Study With Hospital Providers
Source: JMIR Form Res. 2022 Mar 11;6(3):e30946. doi: 10.2196/30946 (PMC8956996; doi:10.2196/30946)
Supplement: Multimedia Appendix 3 [file formative_v6i3e30946_app3.docx]

**Supplementary Table 1.** Example quotes for frequently coded themes.

| **Current suicide risk assessment and interventions** |
| --- |
| *“We have these, kind of, default risk factors that we think [of] from our training. But I think there's so much more that we need to know about all of this.”* |
| ***“****I don’t usually find the PHQ-9 all that helpful...[if] I know they’ve had depression or anxiety in the past, it’s something I’m going to be routinely following up anyway, and I find it more helpful to just start open-ended and say, ‘How are things going?’”* |
|  |
| ***“****I feel like the elephant in the room is that access is so terrible. If I have a patient who's stable and they've been thinking with their therapist, ‘Oh, maybe I should take an antidepressant,’ if my own resources are enough, that's fine. But if it's anything beyond my own personal resources, it is like hell trying to get people help. They go to [the ED] and they may or may not get anything.”* |
| **General attitudes about automated suicide risk prediction algorithms** |
| *“Clinicians aren't always good at knowing if their patients are off track, but giving a little bit of empirical data that sort of charts the patient's trajectory can be really helpful...Particularly, if [the risk algorithm is] kind of including information that we might not exhaustively keep track of over time [such as] chronic health conditions or wounds or something like that.”* |
| *“It strikes me that this could be practice changing in terms of decisions I make as an ER doctor if this is something that works and is built into the system.”* |
| *“I can imagine a situation where someone with a high enough score on this risk prediction tool is triaged to the rapid access psychiatry faster...patients with more resources get seen faster than those who don't have resources, and it sounds like poorer patients might be seen quicker with this kind of tool if it were used effectively.”* |
| *“The key is going to be does it tell me things… about my patients that I didn’t already know or helping me better to stratify them? So I'd probably try it out for a little while. And if it seemed to be adding value, that would be great. If it's not, it would be another alert in [the EHR] that my mind just edits out.”* |
| *“I just feel like the focus on suicide, it makes it automatically more charged, open to a lot of different judgments by a lot of providers which lead to a certain liability concern.”* |
| **Barriers and concerns** |
| *“If we have a quantifiable number and then we didn't hospitalize someone because we didn't think it was therapeutic or we didn't think the acute risk was elevated, and then turns out we were wrong and they did complete suicide...”* |
| *“I tend to...question the veracity of the chart in the EHR. There's so much sort of overpopulating and...information that's just being carried forward without really kind of thinking about it...[like] they had a brother who committed suicide, and that's a significant risk factor. But that may or may not actually be true.”* |
| *“I'd worry about kind of click-fatigue around...if you get kind of this alert every week, okay, you're seeing a high-risk patient, you're like, ‘Yes, I know.’ I could imagine that getting kind of fatiguing and not being super helpful.”* |
| *“There would be a lot of high risk for being misused, like people being sectioned based on a high-risk score or using a cut-off to justify an inpatient stay rather than clinical [judgment].”* |
| *“If I don't have anything I can offer the patient other than, "Here. You can call this number and get on a waiting list," I'd almost rather not know. If this was built into a, yes, when this person has such and such a score on this, there's going to be a response from the mental health division of the hospital...then it would be really useful. But if it's just information that I can't act on, then I'm not sure how useful it is.”* |
| *“You have a 15-minute visit, you're seeing them for whatever problem they're coming in for. You get this notification and then you're scrambling. ‘Well, how am I going to assess them? What if I think they need follow up? How many to get them urgent, it's Friday afternoon,’ –that type of thing.”* |
| **Facilitators and specific recommendations** |
| *“I would hope that the actual criteria the tool had identified would also be available to me so that I could say something like, ‘It has been shown that sometimes when people are dealing with health problems like you are, like X, Y, and Z, that it can really be hard to handle emotionally. And I wonder if it's been difficult for you, and if that's something you want to talk about.’”* |
| *“If their score was a significant amount worse than their previous baseline scores, indicating someone who's acutely worse than they usually are in terms of their suicidality, that would be very helpful to have a sort of pop-up alert.”* |
| *“Why not increase the likelihood that someone is going to follow up? ...there should be someone on the team that is required to kind of process that information somehow to indicate that they read it...if the patient has a psychiatry provider that person is required to click that they read this information. If the person doesn't have a psychiatric provider, then it's the PCP. But my opinion would be, given that information about somebody has had contact with a medical professional, why not have everyone be able to see it so that it increases the likelihood that a person gets additional support or referrals.”* |
| *“I like the push [of the tool] or something that's kind of obvious and hard to opt out of.”* |
| *“I would only want [the tool to be] something that I can go to it and look at it. I don't want anything else shoved in my face...”* |
| **Other factors to consider** |
| *“For me, it's about the timing. Am I going to get that information in a way that I'm able to see it before I see the patient?”* |
| *“My gut answer is that [patients] ought to be able to see the collated information that we see. But...I can imagine there's lots of ethical issues.”* |
| *“Essentially, we're assuming potential bad things in their future. And to have patients see that would - I am sure - bring a lot of concerning questions and gateway messages back, especially if the tool sometimes got it wrong."* |
| *“If it's going to be in the medical record, that medical record belongs to the patient. So if it's going to be in the medical record, it's not for us to decide whether we think they should see it or not. It's there.”* |
